# Zinc(II) Complexes - A New Group of Non-Mutagenic Herpes Simplex Virus Inhibitors

**DOI:** 10.1155/MBD.2000.129

**Published:** 2000

**Authors:** Margarita Pesheva, Tatiana Varadinova

**Affiliations:** 1 Department of Genetics Faculty of Biology Sofia University Sofia 1421 Bulgaria; 2 Laboratory of Virology Faculty of Biology Sofia University Sofia 1421 Bulgaria

## Abstract

Former studies showed that complexes of Zn(II) with picolinic and with aspartic acids, Zn(pic)_2_ and Zn(asp)_2_, are able to inhibit HSV infection in cultured cells by affecting key steps of virus replication. As these complexes are candidates for a novel class anti-HSV drugs, further studies on their mutagenicity are of particular interest. In the present paper we present data showing that Zn(pic)_2_ and Zn(asp)_2_ do not express mutagenic effect in both prokaryotic (*Salmonella typhimurium*) and eukaryotic (*Saccharomices cerevisiae*) test systems.


### ZINC (II) COMPLEXES - A NEW GROUP OF NON-MUTAGENIC HERPES SIMPLEX VIRUS INHIBITORS

Margarita Pesheva[1] and Tatiana Varadinova*[2]

[1] Department of Genetics [2] Laboratory of Virology; Faculty of Biology; Sofia University; 1421 Sofia, Bulgaria. tvar@ns.biofac.uni-sofia.bg, Fax: + 359 2656 641

### Abstract

Former studies showed that complexes of Zn(II) with picolinic and with aspartic acids, $Zn(pic)_2$ and $Zn(asp)_2$, are able to inhibit HSV infection in cultured cells by affecting key steps of virus replication. As these complexes are candidates for a novel class anti-HSV drugs, further studies on their mutagenicity are of particular interest. In the present paper we present data showing that $Zn(pic)_2$ and $Zn(asp)_2$ do not express mutagenic effect in both prokaryotic (*Salmonella typhimurium*) and eukaryotic (*Saccharomices cerevisiae*) test systems.

### Introduction

Systematic studies of the relationship between chemical structure and efficacy or toxicity of metal compounds began around 1931 when Collier and Kraus described the first systematic studies of tumour-inhibiting metal complexes (1). Among metal compounds used in medicine are zinc ones: $ZnSO_4$ is recommenced for the treatment of conjunctivitis, laryngitis and vagynitis, while ZnO is used for topical application against skin diseases. The anti-herpes virus activity of zinc was firstly described by Falke in 1967 (2). Added to Herpes simplex virus (HSV) infected cells, $ZnSO_4$ acts in dose-dependent manner, i. e. concentrations of 0.05 - 0.1mM inhibit the synthesis of HSV proteins (3, 4); 0.2mM suppressed the activity of viral DNA polymerase (3-6), while 15mM decrease the infectious activity of extracellular virions interacting with viral glycoproteins gB and gD (7). Previously reported studies have shown that complexes of Zn(II) with picolinic and aspartic acids are able to inhibit HSV infection in cultured cells by affecting key steps of virus replication (8, 9). As these complexes are candidates for a novel class anti-HSV drugs, further studies on their mutagenicity are of particular interest. In the present paper we present data showing that both $Zn(pic)_2$ and $Zn(asp)_2$ do not express any mutagenic effect.

### Materials and Methods

The mutagenicity of $Zn(asp)_2$ and $Zn(pic)_2$, in concentrations of 100, 10 and 1μM, previously recognised as non-toxic for cultured cells (8, 9) was evaluated. The mutagenicity of zinc compounds was evaluated on both prokaryotic *Salmonella typhimurium*, strains TA100 and TA98 (10), and eukaryotic *Saccharomices cerevisiae*, strains PV2 and PV3 (11) systems. *S. typhimurium* strains are auxotrophic by his and are characterised by gene mutations in *hisG* and *hisD* loci respectively (10). The genotype of the *S. cerevisiae* strain PV2 is:

| *MATa* | *his7-1* | *ade-475* | *lys2-67* | *can1* | *RAD1* |
|---|---|---|---|---|---|
| *MATa* | *his7-1* | *ade-475* | *lys2-68* | *CAN1* | *rad1-5* |

The normal allele of the *RAD1* gene is required for excision repair. The PV3 strain has the same genotype but is homozygous at the *rad1-5* allele (11). As a result of a mutation in *rad1-5* the susceptibility of the PV3 strain to different agents, here including metal compounds, is increased. The prototrophics obtained by reversion of the **ochre** mutations of *his7-1* and mitotic gene conversion in the *lys2* genes were considered. The reversion by his was evaluated using selective medium without his. The mitotic gene conversion was evaluated using selective medium without lys (11). The reversion induced by Zn-compounds in *S. typhimurium* was evaluated without a microsomal fraction S9 (10). Analyses of the induction of the reversions and mitotic gene conversion, as well as of the lethal effect of $Zn(asp)_2$ and $Zn(pic)_2$ in *S. cerevisiae* were evaluated by Inge-Vechtomov *et al.* (11). Cells from the logarithmic phase of growth cultured at 30°C in a liquid YPD (DIFCO) medium with aeration were pelted, washed twice in potassium phosphate buffer (PPB) (pH 7.4) and resuspended in the same buffer until the final concentration of $10^7$-$10^8$ cells/ml. Mixtures of 98 % cell suspension and 2% of Zn-compound in different concentrations were incubated for 4h at 30°C. Compounds were removed by washing in an ice-cold buffer, pelting and resuspending cells in 1ml PPB. The induction of reversions, as well as the mitotic gene conversion were studied on the 5th day after culturing cells at 30°C onto two selective solid media. The viability was evaluated on the 3rd day after culturing at 30°C onto YPD solid medium. Each experiment was in triplicate and the average number of colonies was statistically evaluated (11, 12). A frequency greeter than at least 2-fold over the control background was judged as a significant (13).

**Results and Discussion**

The *his D 305 2* mutation in the *S. typhimurium* strain TA98 is localised in *his D* gene encoding histidine dehydrogenase. This is why TA98 is useful for detecting various frameshift mutagens. The mutation in the other strain, TA100, localised in the *his G-46* gene encoding the enzyme participated in the biosynthesis of histidine and is suitable for detecting mutagens causing G - C base pair substitutions (14).

The fact that under the action of 100μM and 10 μM of $Zn(asp)_2$ and $Zn(pic)_2$ no significant differences in the number of TA98 and TA100 revertants are obtained undoubtedly show that the zinc complexes do not induce any frameshift mutation in the TA98 strain, nor base-pair substitution in the strain TA100. This is why the effect of 1μM was not tested (table 1).

Table 1. Induction of reversions in prokaryotic *S. typhimurium* test system

| | Concentration, in μM | Nr of revertants*: | |
|---|---|---|---|
| | | strain TA98 | strain TA100 |
| $Zn(asp)_2$ | 100 | 49 | 175 |
| | 10 | 55 | 178 |
| Control | - | 54 | 183 |
| $Zn(pic)_2$ | 100 | 54 | 168 |
| | 10 | 54 | 155 |
| Control | - | 55 | 184 |

- as an average Nr of colonies

As far as eukaryotic *S. cerevisiae* cells are concerned, $Zn(asp)_2$ significantly reduced the viability of cells from both tested strains (table 2). No dose-dependent effect nor strain specific response were found.

In contrast, the response of *S. cerevisiae* cells against $Zn(pic)_2$ was strain specific (table 3). Thus, the survival rate of PV2 cells was progressively reduced when the concentrations of the complex decreased. This is well manifested by the fact that 1μM of $Zn(pic)_2$ significantly reduced cell viability as compared to the control. In the concentration range 100-1μM, this complex significantly suppressed the surveillance of PV3 cells as well. The effect was dose-independent and the surviving rate was similar to that obtained after influence PV3 strain with $Zn(asp)_2$.

Table 2. $Zn(asp)_2$ induced reversion and gene conversion in *S. cerevisiae* strains PV2 and PV3.

| Strain | Concentration, in μM | $Ni^{u}$ | Viability, in % | Nr of prototrophic mutants per type* | |
|---|---|---|---|---|---|
| | | | | His x $10^6$ | Lys x $10^5$ |
| PV2 | 100 | 442 | 60.4±2.3 | 2.7±0.08 | 1.7±0.16 |
| | 10 | 497 | 67.9±2.1 | 1.7±0.08 | 3.3±0.14 |
| | 1 | 458 | 62.6±2.3 | 2.3±0.16 | 4.0±0.16 |
| Control | - | 732 | 100.0 | 1.3±0.06 | 2.3±0.16 |
| PV3 | 100 | 444 | 62.4±2.3 | 3.0±0.13 | 5.7±0.16 |
| | 10 | 480 | 67.4±2.1 | 4.3±0.08 | 9.3±0.32 |
| | 1 | 390 | 54.8±2.5 | 4.7± 08 | 10.7±0.18* |
| Control | - | 712 | 100.0 | 2.7±0.08 | 4.7±0.12 |

[u] total Nr of viable cells; * as an average Nr of colonies; $p < 0.05$

In our eukaryotic experimental system, the mutagenic effect of a particular compound is manifested by the appearance of prototrophic colonies, as a result of reversion of *ohre* mutation in the *his7-1* locus. According to Parry (13) the tested compound is recognised as a mutagen when the number of prototrophic colonies is at least two times higher than that in the control. As shown in tables 2 and 3, the number of prototrophic colonies induced by $Zn(asp)_2$ and $Zn(pic)_2$ is not significantly different as compared to the control and show that both zinc complexes did not induce reversion in *his7-1* locus.

$Zn(pic)_2$ did not induce any gene conversion in the *lys2* gene of both PV2 and PV3 strains as well. However, the number of prototrophic PV3 but not of PV2 colonies after action with 1μM $Zn(asp)_2$ was 2.3 times higher than that in the control.

Table 3. $Zn(pic)_2$ induced reversion and gene conversion in *S. cerevisiae* strains PV2 and PV3

| Strain | Concentration, in μM | Ni[u] | Viability, in % | Nr of prototrophic mutants per type* | |
|---|---|---|---|---|---|
| | | | | His x $10^6$ | Lys x $10^5$ |
| PV2 | 100 | 393 | 99.5±0.4 | 1.7±0.08 | 2.7±0.09 |
| | 10 | 324 | 82.0±2.1 | 3.0±0.16* | 3.0±0.16 |
| | 1 | 281 | 71.1±2.7 | 2.3±0.1 | 3.3±0.16 |
| Control | - | 395 | 100.0 | 1.3±0.08 | 2.3±0.08 |
| PV3 | 100 | 531 | 66.4±2.0 | 4.3±0.14 | 6.7±0.15 |
| | 10 | 572 | 71.5±1.9 | 4.7±0.18 | 8.3±0.18 |
| | 1 | 474 | 59.2±2.2 | 5.3±0.2* | 9.7±0.35 |
| Control | - | 800 | 100.0 | 2. ±0.06 | 5.3±0.16 |

[u] total Nr of viable cells; * as an average Nr of colonies; $p < 0.05$

To summarise, the data show that the mutagenicity of a particular zinc complex depends
i) on the specific characteristics of the complex itself: $Zn(pic)_2$ is a mutagen neither for prokaryotic, nor for eukaryotic test systems, while $Zn(asp)_2$ expressed a weak mutagenic effect in eukaryotic *S. cerevisiae* strain PV3 only.
ii) on the specificity of the test system. $Zn(asp)_2$ is not a mutagen for *S. typhimurium* strains, nor for *S. cerevisiae* strain PV2. The sensitivity of the *S. cerevisiae* strain PV3 to 1μM $Zn(asp)_2$ is manifested by a weak mutagenic effect, obviously due to the disturbed reparation system of this strain.

**References**

1. Metal complexes in Cancer and Chemotherapy. B. K. Kuppler (Ed.). VCH, 1993.
2. D. Falke, *Z. Med. Microbiol. Immunol.*, 1967, 153, 175-189.
3. B. Fridlender., N. Chejanovski, Y. Becker. *Virology*, 1978, 84, 551-554.
4. J. Gordon, Y. Asher, Y. Becker. *Antimicrob. Agents Chemother.*, 1975, 8, 377-380.
5. P. Gupta, F. Rapp. *Proc. Soc. Exp. Biol. Med.*, 1976, 152, 455 - 458.
6. J. Shlomai, Y. Asher, Y. G. Gordon, U. Olshevsky, Y. Becker. *Virology*, 1975, 66, 330-335
7. G. Kumel, S. Schrader, H. Zentgarf, H. Daus, M. Brendel. *J. Gen. Virol.*, 1990, 71, 2989-2997.
8. T. Varadinova, I. Pavlov, D. Dimitrov, C. Nachev, P. Bontchev, B. Evtimova, I. Bradvarova, P. Otev. *Comptes Rendus Academ. Bulg. Sci.*, 1990, **11**, 131-133.
9. T. Varadinova, P. Bontchev, C. Nachev, S. Shishkov, D. Strachilov, Z. Paskalev, A. Toutekova, M. Panteva. *J. Chemotherapy*, 1993, **5**, 1, 3-9.
10. D. M. Maron, B. N. Ames, Revised Methods of Salmonella Mutagenicity *Test. Mutat. Res.*, 1983, **113**, 173-215.
11. S. G. Inge-Vechtomov, Y. I. Pavlov, B. N. Noskov, M. V. Repnevskaya, T. S. Karpova, N. N. Khromov-Borisov, J. Chekuolene, D. Chitavichus. Progress in Mutation Research, Geneva: WHO, 1985, **5**, 243-253.
12. Yu. I. Pavlov, N. N. Khromov-Borisov. *Genetica (Moscow)*, 1981, **17**, 1406-1418.
13. J. M. Parry, In: J. Ashby, F. J. de Serres, M. Draper, M. Ishidate, Jr., B. H. Margolin, B. E. Maher, M. D. Shelby (Eds.) Progress in Mutation Research, Vol. 5. Evaluation of Short-Term Tests for Carcinogens. Elsevier, New York, 1985, pp. 25-46.
14. B. N. Ames, In: Hemical Mutagens. Principles and Methods for their Detection. (A. Hollander, ed.). Plenum Press, NY, 1971, vol. 1, pp. 267-282.

**Received: March 30, 2000 - Accepted: May 5, 2000 - Received in publishable format: May 8, 2000**

